# The Measurement of Ethnic and Religious Divisions: Spatial, Temporal, and Categorical Dimensions with Evidence from Mindanao, the Philippines

**DOI:** 10.1007/s11205-015-1145-9

**Published:** 2015-10-22

**Authors:** Omar Shahabudin McDoom, Rachel M. Gisselquist

**Affiliations:** 1 London School of Economics, London, UK; 2 United Nations University – World Institute for Development Economics Research (UNU-WIDER), Helsinki, Finland

**Keywords:** Ethnicity, Ethnic diversity, Ethnic divisions, Fractionalization, Polarization, Segregation, Cross-cutting cleavage, Horizontal inequalities, Mindanao, Philippines

## Abstract

An ever-expanding body of empirical research suggests that ethno-religious divisions adversely impact a host of normatively desirable objectives linked to the quality of life in society, implicitly representing a strong challenge to multiculturalist theory and policies. The appropriate conceptualization and measurement of ethno-religious divisions has consequently become the subject of complex methodological debate. This article unpacks some of this complexity and provides a synthetic critique of how eight key measures each capture the notion of divisions and relate to each other conceptually, theoretically, and empirically within a divided society. It explores simple proportions, fractionalization, polarization, cultural distance, segregation, cross-cuttingness, horizontal inequality, and intermarriage indicators. Furthermore, instead of presenting national-level temporal snapshots of divisions as in much work, it purposely examines how measures also perform at more localized levels of analysis and over time, drawing on individual-level census data from one deeply-divided society, Mindanao, in the Philippines. Analysis underscores four major issues to which researchers should pay more attention: the sensitivity of measures to (1) the underlying causal mechanisms linking divisions with outcomes; (2) the social forces and methodologies shaping the identification and categorization of groups; (3) the passage of time and evolution of divisions; and (4) the level of spatial analysis. The article provides practical guidance and discusses the key implications of these points both for quantitative scholars working with these measures and for qualitatively-inclined empiricists and normative theorists wishing to interpret, evaluate, or otherwise engage the quantitative research on the merits and demerits of diversity.

## Introduction

Nearly 2400 years ago, Aristotle in his description of the ideal *polis* noted an obstacle to the realization of what he called a “choiceworthy life:” “ethnic difference causes faction unless people learn to pull together” (Reeve 1998). Today, a large body of empirical research in political science and economics implicitly challenges the view prominent in multiculturalist policies and normative liberal theory (Kymlicka 1995; Young 1990) that ethno-religious diversity should be promoted and protected in societies by evidencing its deleterious effects on a variety of societal objectives related to the quality of life. Human development (Gerring et al. 2015), social trust (Putnam 2007; Koster 2013), the provision of public goods such as education and health (Alesina et al. 1999; Baldwin and Huber 2010), the absence of violent conflict (Montalvo and Reynal-Querol 2005), democratic transition (Przeworski et al. 2000), and indeed economic growth (Easterly and Levine 1997; Posner 2004; Fedderke et al. 2008) are among the outcomes an ever-expanding body of empirical work suggests ethnic diversity adversely impacts. The appropriate measurement of ethno-religious divisions has consequently become the subject of rich discussion in the scholarly literature. To capture these divisions in their analyses, many researchers have commonly used, and continue to use, the index of ethno-linguistic fractionalization (ELF). A number of researchers, however, have noted serious limitations with this indicator, and the growing debate among them on how best to measure such divisions underscores the significant methodological complexity in this area.^1^

This paper contributes to this debate and unpacks some of its complexity by exploring how different measures of ethno-religious divisions relate to each other conceptually, theoretically, and empirically within the context of a divided society. Furthermore, while much of the extant literature has focused, largely due to data constraints, on measuring these divisions at the national level and on a single snapshot in time, we purposely make our focus the spatial and temporal dimensions of the measures and explore here how divisions manifest at more localized levels and how they evolve over time. We draw in this analysis on individual-level census data for Mindanao, a deeply divided society and the second-largest island group in the Philippines, with a population of 21.9 million (2010 Census). These data allow for greater empirical precision and deeper theoretical insight into ethnic divisions than permitted using more aggregate statistics. The article focuses on eight measures from the literature on ethnic politics, including those most commonly used, plus several more that warrant consideration and are relevant to the examination of major theories: simple proportions, fractionalization, cultural (distance) fractionalization, polarization, segregation, intermarriage, horizontal inequality, and cross-cuttingness. We consider both ethnic and religious cleavages for all measures, and we explore how each measure varies across multiple levels of analysis (Mindanao, region, province, municipality, and barangay) and over time (2000 and 2010).

The paper has three readerships in mind. First, for those interested in divided societies, it evidences in one case the complex, multi-faceted, and dynamic character of ethno-religious divisions and the challenge of conveying them. Second, for scholars working on how ethnic and religious politics influence various dimensions of the quality of life, it serves as a synthetic and comparative guide to how divisions are conceptualized and measured in quantitative work across their respective literatures. Third, for qualitatively-inclined empiricists and normative theorists wishing to interpret, evaluate or otherwise engage the quantitative research on the merits of diversity, it highlights four conceptually “big” issues that, we argue, deserve more attention and further investigation. These four issues will not be altogether new to specialists of ethnic politics measurement, but when compared with previous work, the fine-grained data here allow for more precise examination of each and for new insights into related methodological and theoretical points:

1. *Sensitivity to the choice of measure* Measures of divisions rely on distinct theoretical logics, and, unsurprisingly, do not all correlate well empirically. As scholars, we should take care to select our measures with explicit reference to theory and specifically their fit with posited mechanisms. One underestimated concern is the potential mismatch between the individual-level logics used in several prominent mechanisms and the aggregation logics implicit in all the measures.
2. *Sensitivity to categorization* The decision of which ethno-religious categories to use in measurement is highly consequential yet rarely obvious, even in divided societies. As group identities are fluid and socially-constructed, it is important then to illuminate the forces at work and to justify the categorization methodology behind group classification. Nation-building and minority promotion policies for instance may powerfully shape the motivations of state officials and incipient social groups respectively.
3. *Sensitivity to time* Divisions evolve and so *when* data is collected matters; substantial changes in some of these measures can be seen even within a relatively brief period such as a decade. Using outdated data—as is often done—thus is problematic. We then should pay more attention to the drivers of change—migration, modernization, and assimilation for instance—in the societies that we study.
4. *Sensitivity to space* Divisions manifest differently at different levels of analysis and researchers should accordingly beware the modifiable areal unit problem and the ecological fallacy. We should identify the appropriate level of analysis using theory to specify the mechanism or causal pathway through which ethnic divisions lead to observed outcomes—not, as is often done, simply conduct analysis at the lowest level of aggregation for which we can obtain data. We should also be more sensitive to how spatial organization, notably settlement patterns, influences societal divisions.

The next section of this article unpacks the conceptual complexity of the notion of an “ethnic division” and compares the conceptualizations implicit in each of the eight measures. The article then introduces Mindanao, the divided society for which we explore the measures, before turning to discussion of how the eight measures capture its divisions empirically. Finally, the article synthesizes the empirical discussion to substantiate the four points listed above before concluding with discussion of implications for future research.

## A Menu of Measures

Researchers interested in the effects of ethnic diversity and division on quality of life outcomes face a myriad of quantitative measures.^2^ This article focuses on eight of the key ones: simple proportions, fractionalization, cultural (distance) fractionalization, polarization, segregation (index of dissimilarity), intermarriage (intermarriage index), horizontal inequality (group-weighted coefficient of variation—GCOV), and cross-cuttingness. While a variety of other measures exist, these are the ones most commonly used in the quantitative literature on ethnic politics and political economy, plus several others we believe warrant more consideration given their relevance to major theories. Mathematical formulae are summarized in Table 1.

**Table 1:** Diversity measures

Simple proportions	*p*_*i*_	Where *p*_*i*_ is the proportion of individuals who belong to group *i*
Fractionalization (Taylor and Hudson 1972)	$1 - \mathop \sum \limits_{i = 1}^{n} p_{i}^{2}$	Where *p*_*i*_ is the proportion of individuals who belong to group *i* and *n* is the number of groups
Cultural (distance) fractionalization (Fearon 2003)	$1 - \mathop \sum \limits_{i = 1}^{n} \mathop \sum \limits_{j = 1}^{n} p_{i} p_{j} r_{ij}$	Where *p*_*i*_ is the proportion of individuals who belong to group *i*, *p*_*j*_ is the proportion of individuals who belong to group *j*, *n* is the number of groups, and *r*_*ij*_ is a measure of cultural distance (linguistic similarity) between groups *i* and *j* ^a^
Polarization (Montalvo and Reynal-Querol 2005)	$1 - \mathop \sum \limits_{i = 1}^{n} \left( {\frac{{0.5 - p_{i} }}{0.5}} \right)^{2} p_{i}$	Where *p*_*i*_ is the proportion of group *i* and *n* is the number of groups
Segregation (index of dissimilarity) (Duncan and Duncan 1955)	$0.5 \mathop \sum \limits_{i = 1}^{n} \left| {\frac{{ m_{i} }}{M} - \frac{{ c_{i} }}{C}} \right|$	Where *m*_*i*_ and *c*_*i*_ are the populations of each group respectively in the smaller geographic sub-unit, *i*, and *M* and *C* are the total populations of each group in the larger geographic unit under analysis, and *n* is the number of geographic sub-units
Intermarriage index (Schoen 1988)	$\frac{{\frac{{C_{ij} }}{{M_{i} }} + \frac{{C_{ji} }}{{F_{i} }} + \frac{{C_{ji} }}{{M_{j} }} + \frac{{C_{ij} }}{{F_{j} }}}}{{\frac{{(C_{ii} + C_{ij} )}}{{M_{i} }} + \frac{{\left( {C_{ii} + C_{ji} } \right)}}{{F_{i} }} + \frac{{(C_{jj} + C_{ji} )}}{{M_{j} }} + \frac{{(C_{jj} + C_{ij} )}}{{F_{j} }}}}$	Where *M*_*i*_ is the size of the male population (married and unmarried) of group *i*, *F*_*j*_ is the size of the female population (married and unmarried) of group *j*, and *C*_*ij*_ is the number of unions between males of group i and females of group *j*
Horizontal inequality (GCOV) (see Mancini 2005)	$\frac{1}{{\bar{y}}}\left( {\mathop \sum \limits_{r}^{R} p_{r} \left( {\left( {\bar{y}_{r} - \bar{y}} \right)^{2} } \right)} \right)^{{\frac{1}{2}}}$	Where *y* is the quantity of the variable of interest (e.g., level of education); $\bar{y}_{r} = \frac{1}{{n_{r} }}\mathop \sum \nolimits_{i}^{{n_{r} }} y_{ir}$ and is the mean value of *y* for group *r*; *R* is the number of groups; and *p*_*r*_ is group *r*’s population share
Cross-cuttingness (Selway 2011)	$$1 - \sqrt {{\raise0.7ex\hbox{${\mathop \sum \nolimits_{i = 1}^{r} \mathop \sum \nolimits_{j = 1}^{c} \frac{{\left( {O_{ij} - E_{ij} } \right)^{2} }}{{E_{ij} }}}$} \!\mathord{\left/ {\vphantom {{\mathop \sum \nolimits_{i = 1}^{r} \mathop \sum \nolimits_{j = 1}^{c} \frac{{\left( {O_{ij} - E_{ij} } \right)^{2} }}{{E_{ij} }}} {Nm}}}\right.\kern-0pt} \!\lower0.7ex\hbox{${Nm}$}}}$$	Where $$\mathop \sum \limits_{i = 1}^{r} \mathop \sum \limits_{j = 1}^{c} \frac{{\left( {O_{ij} - E_{ij} } \right)^{2} }}{{E_{ij} }}$$ is the Chi square test statistic in a contingency table; *r* is the number of rows (e.g., number of ethnic groups); *c* is the number of columns (e.g., number of religious groups); *O*_*ij*_ is the actual number in cell_*ij*_; *E*_*ij*_ is the expected number in cell_*ij*_; *N* is the sample size; and *m* is the smaller of either (*r* − 1) or (*c* − 1). It is Cramer’s V subtracted from 1.

^a^ $r_{ij} = \frac{l}{15}^{\alpha }$ where *l* is the number of shared classifications between *i* and *j,* 15 is the maximum number of classifications in the dataset, and *α* is set at ½. Alternatively, for instance, Desmet et al. (2009) set it at 1/20

A handful of key conceptual aspects of ethnic divisions are captured in these measures. Each measure in turn suggests an underlying conceptual logic and link to theories of ethnic politics and political economy. Such links are sometimes explicit, but more often implicit. While there is not space here to review the conceptualization and operationalization of each measure in depth, much less all of the ways in which each might be linked to relevant theoretical literatures, we introduce key conceptual aspects captured in these measures and discuss some of the links to major theories. Which of these measures should researchers then use? As discussed in the final section of this paper, the answers here should vary, based on careful consideration of which measures best fit mechanisms underlying theoretical hypotheses. Further, it should be noted that some key conceptual aspects highlighted in theory are not captured in any of these measures, suggesting the need for continued work on measurement.

The key aspect highlighted in each of the eight measures is an accounting for *social structure*, specifically the number and sizes of groups.^3^ The complex configurations possible on these two structural dimensions support manifold theoretical logics. For instance, as the number of groups in society rises, coordination between groups may be more difficult, making collective action less likely and conflict more likely (Hardin 1995). Alternatively, as a group increases in size, it may become more threatening within society. Thus, one theory of ethnic civil war assumes that societies comprising two equally-sized groups will also be maximally-polarized (Montalvo and Reynal-Querol 2005). The logic of both group number and group size also underpins theories of electoral mobilization where political elites calculate the support needed to achieve a minimum winning coalition (Riker 1962).

The *dimensionality* of divisions is another key aspect captured in these measures. The first six measures listed above are one-dimensional, capturing only one ethnic dimension at once. The last two, horizontal inequality and cross-cuttingness, are two-dimensional, providing information about two social dimensions at once (e.g., ethnicity and socioeconomic class, or language and religion).^4^ While non-specialists often treat ethnic divisions—and social divisions more broadly—as relatively straightforward and singular, the research literature shows clearly that in fact they tend to be multi-dimensional, based both in multiple ascriptive characteristics such as tribe, race, language, and caste (often collectively equated with a broad conceptualization of ethnicity) and in more attitudinal characteristics such as class, ideology, and religion (Lane and Ersson 1994). In this sense, all measures reviewed here potentially underestimate the extent of a society’s divisions.

In using these measures in analysis, the multidimensionality of societal divisions can be addressed in part through inclusion of several one-dimensional measures calculated for multiple dimensions individually—e.g., the ELF for both ethnicity and religion. However, this fix does not fully address the interrelations between dimensions. Consider, for instance, the conceptual aspects and theoretical discussions that can be assessed using the two-dimensional measures, cross-cuttingness and horizontal inequality. Classic theories suggest that “reinforcing” cleavages imply deeper division and more conflict (Lijphart 1977). Likewise, the coincidence of ethnicity and class in “ranked” societies—which can be assessed using measures of horizontal inequality—features in major theories of ethnic conflict (Horowitz 1985). By contrast, cross-cutting cleavages—where characteristics cut across, rather than fall along, group boundaries—are seen to moderate divisions. A society comprising two ethnic groups each in turn comprising equal proportions of Protestants and Catholics would have a cross-cutting cleavage. The logic of cross-cutting cleavages lies at the heart of theories of political stability in multi-party and multiethnic democracies, for example. In such societies, political attitudes and beliefs are expected to be less intense because individuals feel “cross-pressured” or pulled between conflicting forces (Lipset and Rokkan 1967).

It is not only the existence of different groups of different sizes but also the *intensity* of their differences that may matter: greater differences signify deeper divisions. Early theories of genocide, for instance, emphasized deep cleavages within society (Kuper 1982). The notion is closely related to the constructs of *social distance* (Bogardus 1933) and *identity salience* (Brewer 1985). Yet conceptualizing a division’s intensity or the distance between groups is challenging. The extent of difference may be more a matter of subjective experience than objective quantification. Hutu and Tutsi in Rwanda share language, culture, and religion but nonetheless feel their differences very strongly. Cultural (distance) fractionalization, for one, speaks to this aspect of division by using data on language families to assess the cultural distance between ethnic groups based on their predominant language. Horizontal inequality and cross-cuttingness measures also speak to distance by capturing observed differences in socioeconomic status and other social markers, respectively.

*Status asymmetries*, such as those captured by the horizontal inequality measure, may motivate the negative sentiments that characterize divided societies. High status groups may feel contempt and low status groups resentment vis-à-vis each other. This asymmetry may be material. Economic inequality and political exclusion along ethnic lines, for instance, have featured in explanations of ethnic civil wars (Cederman et al. 2011). Similarly, relative deprivation, a construct operational at both the individual and group-levels, has been linked to the broader notion of rebellion (Gurr 1970). The conceptual logic linking theories based on material asymmetry is grievance. Yet the asymmetry may also be symbolic. The theoretical logic emphasized in this context is threats to, or anxiety over, a group’s self-esteem. Such perceptions are believed also capable of motivating ethnic conflicts (Horowitz 1985).

Ethnic division may further be observed in the *spatial organization* of groups, as captured by segregation measures, another conceptually important aspect of ethnic cleavages. Settlement patterns may indicate a preference to live with co-ethnics and may also affect interaction within and across ethnic groups. We would expect a society comprising two equally-sized groups living in two territorially-distinct regions to function differently to a society where members of the same two groups live side-by-side as neighbors. The spatial organization of groups is also relevant to institutional theories of governance and democracy in plural societies. The design of consociational or majoritarian institutions, for instance, may turn on the geographic distribution of ethnic groups (Lijphart 1977).

Segregation and other measures in turn may be suggestive of levels of *interaction* between groups but, of the measures explored here, only intermarriage is based directly on observed behavior. Interaction and cross-group ties are believed to diffuse information, build trust, and facilitate cooperation across social boundaries. The logic is conceptually embedded in social capital theory (Putnam 2000) and in theories of interethnic conflict and cooperation (Fearon and Laitin 1996; Varshney 2001) where connections that bridge ethnic networks are seen as desirable. The broader notion of intergroup “contact,” developed within social psychology, is conceptually related and is believed, under certain conditions, to reduce prejudice between groups (Allport 1958; Hewstone and Swart 2011).

Table 2 summarizes which of the above conceptual logics underpins each measure. It is also worth noting that some aspects of ethnic divisions highlighted in the theoretical literature are not captured by any of these measures. For instance, with reference to the last point, none of these measures is based on observations about the type of behavioral interaction specifically highlighted in Putnam (2007)’s and Varshney (2001)’s work—through formal associations of civil society. Neither does any measure capture the extent to which state institutions such as censuses, ethnic quotas, or intermarriage laws create or maintain ethnic boundaries (Lieberman and Singh 2012). Nor do any speak to the internal *organizational strength* or cohesiveness of groups themselves, which may in turn influence their ability to mobilize (Van Cott 2007). Relatedly, no measure distinguishes between elite and mass-based divisions, although some theories suggest elite behavior (e.g., inter-elite bargains) may matter more for coexistence in plural societies than mass sentiments (Lijphart 1977). Nor, as we return to below, do these measures capture temporal issues such as changes in diversity over time or temporal anteriority, the notion that “we were here before you.” Yet these time-related issues feature prominently in theories of immigrant and indigenous politics for instance.

**Table 2:** Conceptual aspects of measures compared

	Simple proportions	Fractionalization	Cultural (distance) fractionalization	Polarization	Segregation (index of dissimilarity)	Intermarriage index	Horizontal inequality (GCOV)	Cross-cuttingness
Number and/or size of groups (social structure)	✓	✓	✓	✓	✓	✓	✓	✓
Multi-dimensional cleavages recognized							✓	✓
Division intensity/social distance between groups			✓				✓	✓
Interaction between groups					✓	✓		
Spatial organization of groups					✓			
Status asymmetry between groups							✓	
Organizational strength and cohesiveness of groups								
Elite and mass-based divisions distinguished								
Temporal change								

## Case Selection

Mindanao, the southernmost of the three main island groups that make up the Philippines, is home to approximately 22 million individuals who together comprise a society remarkable for the multiplicity, complexity, and depth of its divisions. Mindanao then represents a particularly hard case for any single measure that seeks to capture its divisions and this in part motivated its selection. A single case selected for illustrative purposes necessitates cautious generalization. Nonetheless we believe the methodological upside is analytical depth and empirical precision and these make the trade-off with generalizability less unattractive.

Historically, Mindanao has followed a different trajectory to the rest of the Philippines in large part due to its early encounter with Islam in the fifteenth century (Majul 1973). Centralized Islamic rule quickly took root in the region, most prominently in the form of the sultanates of Sulu, Maguindanao, and Buayan. In contrast, the two more northern Philippine island groups of Luzon and Visayas had stronger contact with Christianity through Spanish colonial conquest starting in the late sixteenth century. The Moro, the collective identity of Mindanao’s Muslim ethnic groups, fought a long series of wars, spanning more than 300 years, against Spanish annexation. When Spain finally lost the Philippines to the United States in 1898, Mindanao’s new colonizer came to govern the territory through accommodations reached with certain local Muslim elites (Abinales 2010). Moro resistance re-emerged, however, following independence in 1946, this time against incorporation into the modern Filipino nation-state. Mindanao is also home to the Lumad, the collective identity of the island’s mostly un-Islamicized and un-Christianized indigenous groups. As a smaller, less organized minority, their situation, although similar in several ways to that of the Moro, has garnered much less attention.

The mass migration of Filipino Christian settlers from the Luzon and Visayas island groups, instituted under American rule and expanded by the post-independence Philippines government, dramatically restructured Mindanao’s demography and lies at the heart of its native-settler conflict. Mindanao’s Muslim population, which today numbers nearly 5 million, declined from 76 to 22 % between 1903 and 2010 and the Lumad, who comprise over 3 million individuals today, experienced a similar minoritization. Mandatory land registration, also introduced during the American administration, compounded the Moro and Lumad sense of dispossession as it allowed the ownership of many ancestral lands to pass into foreign, often settler, hands. Today, the provinces in which Mindanao’s Muslims are concentrated have some of the worst poverty, education, and health indicators in all of the Philippines (McKenna 1998). Moro, and Lumad, attribute their marginalization to indifference if not discrimination from the Philippines central government. In part, however, the subordinate position of ordinary Moro can be traced to local leadership. Local Muslim lords (*datus*), even today, wield considerable influence over many ordinary Moro through strong clientelist bonds and engage in bitter and sometimes violent inter-clan rivalries (*rido*) that represent in their own right another important societal divide (Torres 2014).

The indigenous population’s sense of historical injustice and contemporary disadvantage has fuelled communal violence and motivated several armed Moro separatist movements since independence. Mindanao’s civil war, at its peak between 1972 and 1976, saw the rise of both the Moro National Liberation Front (MNLF) and the idea of an independent Bangsamoro (Moro Land) (McKenna 1998). The war killed, by one estimate, 50–100,000 individuals and displaced a million more (Ahmad 2000). It remains not fully resolved today in part due to differences between the separatist movements. An initial agreement with the MNLF that would eventually create the Autonomous Region of Muslim Mindanao (ARMM) in 1991 was shunned by its main breakaway rival, the Moro Islamic Liberation Front (MILF). A subsequent agreement with the MILF, in 2014, to replace ARMM with the “Bangsamoro Political Entity,” in turn alienated elements within the MNLF. While lasting peace remains uncertain, violence and displacement have continued and deepened Mindanao’s divisions.

## The Data

We draw on data from the 2000 and 2010 censuses for the Philippines. Unusually for a national census, the Philippines National Statistics Office released individual-level records for *all* households in Mindanao providing us with an extraordinarily rich source of information: our dataset contains detailed information on 21.9 million individuals in 2010 and 18.1 million individuals in 2000. As there had been administrative boundary changes in the 10-year interval, we realigned the 2010 data to make them comparable with the 2000 data.^5^ The Philippines’ territorial organization comprises four administrative levels and in 2000 Mindanao was composed of 6 regions, 25 provinces, 430 municipalities, and 10,019 barangays. We constructed the various measures of divisions for all administrative levels and for both census years.

Following recent literature, we focus our analysis on *politically salient* ethno-religious categories to capture societal divisions (Posner 2004; Mozaffar et al. 2003; Wucherpfennig et al. 2011). As the case analysis above suggests, two dimensions of ethno-religious cleavage have been particularly salient in Mindanao politics.^6^ The first, which we label “ethnic” for simplicity, includes three categories: Moro, Lumad, and Settler. The second dimension, religion, includes three salient religious affiliations: Christian, Muslim, and Other. We detail the process of how we recoded the census categories to capture these two dimensions in the section below related to categorization sensitivity.

### The Measures Applied: Mindanao in Comparative Perspective

Applied to Mindanao, the eight measures we examine collectively illustrate the complex, dynamic, and multi-faceted nature of ethnic divisions in a divided society. Below we discuss what is learned from each of these measures in turn, comparing Mindanao’s scores against those in other countries and regions when available. Overall, the measures broadly confirm that Mindanao is, comparatively, a deeply-divided society. It is highly fractionalized and polarized, strongly spatially and socially segregated, and has ethnically and religiously reinforcing cleavages. However, as we will see, deeper analysis suggests a more complex portrait: measures describe generally higher levels of religious and ethnic division at higher administrative levels than at lower levels; furthermore, depending on the measure chosen, they suggest either increasing or decreasing divisions between 2000 and 2010. Table 3 provides illustrative mean values for all eight measures in 2000 and 2010 across the five administrative levels.^7^

**Table 3:** Indicators of ethnic and religious divisions compared across time and space (mean values)

Indicator	Mindanao (n = 1)	Region (n = 6)	Province (n = 25)	Municipality (n ≤ 430)^a^	Barangay (n ≤ 10,019)^b^
*Simple proportions*					
Ethnicity (Moro)					
2000	0.20	0.24	0.25	0.26	0.30
2010	0.22	0.26	0.27	0.27	0.31
Religion (Muslim)					
2000	0.21	0.24	0.26	0.26	0.30
2010	0.22	0.26	0.27	0.27	0.31
*Fractionalization (0* = *minimum; 1* = *maximal)*					
Ethnicity					
2000	0.46	0.30	0.28	0.20	0.12
2010	0.53	0.37	0.34	0.24	0.16
Religion					
2000	0.37	0.20	0.17	0.13	0.08
2010	0.38	0.22	0.17	0.13	0.08
*Cultural (distance) fractionalization (0* = *minimum; 1* = *maximal)*					
Ethnicity					
2000	0.44	0.29	0.27	0.19	0.12
2010	0.49	0.36	0.33	0.23	0.15
Religion					
2000	0.18	0.10	0.08	0.06	0.04
2010	0.18	0.10	0.08	0.06	0.04
*Polarization (0* = *minimum; 1* = *maximal)*					
Ethnicity					
2000	0.75	0.55	0.51	0.37	0.23
2010	0.81	0.67	0.62	0.44	0.30
Religion					
2000	0.68	0.38	0.32	0.24	0.15
2010	0.71	0.41	0.33	0.25	0.16
*Segregation (index of dissimilarity) (0* = *minimum; 1* = *maximal)*					
Ethnicity (Moro:non-Moro)					
2000	0.88	0.76	0.72	0.64	n/a
2010	0.86	0.70	0.66	0.58	n/a
Religion (Muslim:non-Muslim)					
2000	0.88	0.76	0.73	0.65	n/a
2010	0.87	0.73	0.70	0.61	n/a
*Intermarriage index (0* = *always inmarry; 0.5* = *indifferent; 1* = *always outmarry)*					
Ethnicity (Moro:Settler)					
2000	0.01	0.04	0.05	0.12	0.14
2010	0.01	0.05	0.06	0.16	0.19
Religion (Muslim:Christian)					
2000	0.01	0.05	0.05	0.13	0.12
2010	0.01	0.06	0.07	0.16	0.15
*Horizontal inequality (GCOV and education) (0* = *minimum; 1* = *maximal)*					
Ethnicity					
2000	0.15	0.12	0.12	0.10	0.09
2010	0.14	0.11	0.11	0.09	0.08
Religion					
2000	0.09	0.07	0.08	0.06	0.06
2010	0.09	0.06	0.07	0.05	0.05
*Cross*-*cuttingness (0* = *perfectly reinforcing; 1* = *perfectly cross*-*cutting)*					
Ethnicity and religion					
2000	0.31	0.38	0.42	0.58	0.54
2010	0.30	0.36	0.40	0.52	0.50

We calculate the measures for each subnational unit using the same three religious (Muslim, Christian, Other) and same three ethnic groups (Moro, Settler, Lumad) that exist at the national level. Consequently, for the intermarriage, segregation, horizontal inequality, and cross-cuttingness measures, we exclude those subnational units where any one of these groups was entirely absent (i.e. had zero members). To include such subnational units would generate values with potentially misleading implications for our analysis

^a^At the municipal level, n is lower for four measures due to missing data arising primarily from the decision described above: for the intermarriage index, n = 419 in 2000 and n = 423 in 2010 for religion; for horizontal inequality, n = 402 for ethnicity and 403 for religion in 2000 and n = 428 and 372, respectively, in 2010; for cross-cuttingness, n = 405 for 2000 and 2010; and for segregation, n = 427 and 417 in 2000 for ethnicity and religion respectively and n = 428 and 429 in 2010 for ethnicity and religion respectively

^b^At the barangay level, n is lower for various measures due to missing data arising in part from the decision described above: for simple proportions, n = 10,015 for ethnicity and 10,019 for religion; for fractionalization, cultural (distance) fractionalization, and polarization, n = 10,015 for ethnicity in 2000 and n = 10,019 for all others; for the intermarriage index, n = 4215 and 5245 for religion in 2000 and 2010, and n = 4810 and 5276 for ethnicity; for horizontal inequality, n = 3263 for ethnicity and 2623 for religion in 2000, and n = 5128 and 3402, respectively, in 2010; for cross-cuttingness, n = 2145 in 2000, and n = 3224 in 2010

### Simple Proportions

The size of each ethnic or religious group relative to the total population is an intuitively-understood and readily-calculated metric. In terms of ethnicity, the 2010 census shows the Mindanao population to be 63.44 % Settler, 22.28 % Moro, and 14.28 % Lumad, while in terms of religion, it is 75.39 % Christian, 22.05 % Muslim, and 2.55 % Other.^8^ Such figures are broadly comparable to those reported in several divided societies, such as Bolivia (64 % indigenous, 25 % mestizo, 10 % white) and Israel (79 % Jews, 18 % Palestinian, 3 % Druze or unclassified), but divisions also exist in societies with different demographic compositions (Alesina et al. 2003a).

A major challenge in employing simple proportions is that there is little consensus on their precise interpretation. For instance, based on the region’s political history, we might predict the size of the Muslim/Moro population to be especially salient to political outcomes in Mindanao. As Table 3 shows, Muslims and Moros each represented about one-fifth of the population in 2000 and 2010, with mean values increasing monotonically to an average of roughly 30 % at lower administrative levels. One interpretation then might be that Muslim/Moro political influence should be higher at lower administrative levels. An alternative perspective based on the notion of a minimum winning coalition would emphasize instead the size of the larger populations: both Settler and Christian groups each constitute winning coalitions of larger than 50 % each on average at all administrative levels. Strategically then, political entrepreneurs might achieve majority support by focusing on either of these constituencies and ignoring Muslim and Moro constituencies (see Riker 1962).

### Fractionalization

The index of ethno-linguistic fractionalization (ELF) has prevailed as the most commonly-used measure of ethnic divisions in quantitative work. Intuitively, it describes the probability that two individuals selected at random will be from different ethnic groups and ranges from complete homogeneity (0) to complete heterogeneity (1). The measure is an adaptation of the Herfindahl–Hirschman index, a measure of market concentration (Herfindahl 1950; Hirschman 1945).^9^ Its primacy is partly attributable to its early adoption in the literature. Taylor and Hudson (1972)’s calculation of the ELF, based primarily on data compiled in 1960 in the Soviet *Atlas Narodov Mira* (1964), was used in several early studies exploring heterogeneity and growth, including Mauro (1995) and Easterly and Levine (1997). Later work updated these data (Roeder 2001) and calculated the ELF using other cross-national sources (Alesina et al. 2003b). In Alesina et al. (1999), fractionalization measures calculated using national data have also been used as a proxy for the polarization of preferences.

Cross-national data on ethnic fractionalization shows a world average of 0.44 (Alesina et al. 2003a). As Table 3 shows, ethnic fractionalization values in Mindanao have been slightly higher that this global average, but decline as we move towards the barangay level, suggesting that smaller communities are more ethnically homogeneous. Fractionalization values are also higher for ethnicity than religion, and higher in 2010 as compared to 2000, suggesting divisions increasing over time.

### Cultural (Distance) Fractionalization

Many standard diversity measures treat all differences between ethnic groups as equivalent, but some differences may be more meaningful that others. In particular, the depth of linguistic and cultural differences between groups may play a role, for instance, in hindering inter-group interaction or may proxy for greater differences in inter-group preferences. The most commonly used measure that takes cultural distance into account is cultural fractionalization, which is fractionalization weighted by cultural distance (Greenberg 1956; Fearon 2003). Cultural distance is assessed based on language and derived from the number of shared language tree branches between the dominant or common language of each ethnic group.^10^

Consistent with Fearon’s analysis at the cross-national level, Mindanao’s cultural fractionalization values (Table 3) tend to be slightly lower than standard fractionalization values, and, as with Mindanao’s fractionalization values, generally lower at lower administrative levels, suggestive of the geographic segregation of ethno-religious groups. Religious groups in Mindanao are not well distinguished by language differences, so cultural fractionalization along religious lines is lower than that for ethnicity.

### Polarization

Ethnic polarization is a demographic measure that assumes a society is most deeply-divided when it comprises two groups each representing half of the population (Reynal-Querol 2002). The measure’s underlying logic, adapted from Esteban and Ray (1994), assumes group members share characteristics that differ from other groups’ characteristics and that societal conflict is most likely when these characteristics are distributed bimodally in a population.

Polarization scores for Mindanao (Table 3) reveal an empirical pattern similar to fractionalization: As the administrative level declines, polarization declines. At the Mindanao-level, society appears highly polarized ethnically with a score of 0.75 in 2000, comparable to Sri Lanka. At the barangay-level, ethnic polarization is much lower with a mean of 0.23, comparable to Austria.

### Segregation

Conceptually, segregation can be thought of as the “extent to which individuals from different groups occupy and experience different social environments” (Reardon and O’Sullivan 2004). The construct has served in scholarly analysis both as *explanandum* and *explanans* where it has been associated with poor outcomes in poverty, health, education, and crime in the U.S. urban context. Segregation may also be the result of ethnic violence or of explicit policies of discrimination.

We present perhaps the most widely-used measure of segregation, the index of dissimilarity (Duncan and Duncan 1955). Technically, dissimilarity measures “the proportion of minority members that would have to change their area of residence to achieve an even distribution” within the region under analysis (Massey and Denton 1988).^11^ The dissimilarity index then emphasizes “evenness” in settlement patterns.^12^ In Mindanao, the index of dissimilarity between Muslims and non-Muslims (and between Moro and non-Moro) is high at all administrative levels (see Table 3). Segregation is adjudged severe in the U.S. context when the index attains 0.80 and higher. In Mindanao as a whole the score for Muslim/non-Muslim segregation is 0.88 in 2000. As with other measures, these values decline as the administrative level declines, but their decrease is less dramatic in relative terms than for the other measures.^13^ The fact that divisions also appear less severe using other measures at lower levels of government is suggestive of the spatial segregation of ethno-religious groups at higher levels of analysis.

### Intermarriage

Intermarriage is shaped by both individual preferences and structural opportunities to outmarry (Kalmijn 1998). Preferences may reflect an individual’s desire for particular socio-cultural resources in a partner as well as group norms and sanctions regarding exogamy. Insofar as preferences matter then, intermarriage is likely a good indicator of societal divisions as it is a direct measure of behavioural interaction across group boundaries. Opportunity factors, however, notably the relative sizes and the spatial organization of groups, also affect intermarriage rates. Individuals from small minority groups may have to outmarry and individuals from groups that are geographically isolated may have to inmarry. The intermarriage index, first developed by Schoen (1988), addresses the confounding issue created by relative group size. The index is scaled from 0 to 1 where 0 indicates perfect endogamy and 1 perfect exogamy. A value of 0.5 indicates random selection of marital partner. Despite its relevance to the study of ethnicity, quantitative work in political science and political economy has rarely used measures of intermarriage, while it is more commonly used in sociology. A key practical constraint is the need for individual-level or household-level data to calculate intermarriage rates.

In Mindanao, intermarriage is rare, but interestingly, increased between 2000 and 2010. Assuming that higher intermarriage reflects better intergroup relations, this suggests Mindanao may be becoming less divided, which on the surface appears different to the story told by fractionalization and polarization measures. Intermarriage rates are also higher at lower administrative levels. This appears broadly consistent with what we see in other measures: communities appear less divided at lower levels of analysis.

Finally, it is worth noting the reduced sample size at the municipal and barangay levels. This is due to the perfect homogeneity of these communities: they contain zero members of certain ethnic and religious groups that are salient at the Mindanao level. As a consequence, the measure cannot be calculated as it would involve the impossibility of dividing by zero, suggesting a potential limitation to the use of the measure at subnational levels in spatially segregated societies.

### Horizontal Inequality

Another characteristic of ethnic division is the degree of economic, political, and social inequality between members of different ethnic groups—i.e., horizontal inequality (Stewart 2008). Cross-national analyses suggest that horizontal inequalities between ethnic groups—more than ethnic difference, cultural distance, or income inequality alone—may drive observed relationships between ethnic divisions and both ethnic civil war (Cederman et al. 2011) and low public goods provision (Baldwin and Huber 2010).

Horizontal inequality has been addressed in the literature in multiple ways, but no consensus has emerged in quantitative analyses on a single preferred measure. Here we focus on the coefficient of variation by groups (GCOV), a common measure of regional disparities and among the three measures favored by Stewart (2008).^14^ We focus on socioeconomic inequality, which in the absence of income or employment data in the census is assessed using available data on educational attainment. The data are grouped to create a 10-point scale, where 1 indicates no education and 10 indicates education beyond the baccalaureate degree.

As shown in Table 3, GCOV at the Mindanao level is between 0.14 and 0.15 for ethnic groups and about 0.09 for religious groups.^15^ It further declines for both types of groups at lower administrative levels. Like the intermarriage index, we focus here only on heterogeneous communities in which all ethnic and religious groups salient at the national level are present; thus, the sample size is reduced at lower administrative levels, notably municipalities and barangays.^16^

The figures here appear broadly comparable to those reported by Mancini (2005) in similar analysis across Indonesian districts using years of education (with average GCOV of 0.10). It is worth noting that a key challenge in considering horizontal inequality remains the relative lack of comparable, cross-national data. This stems both from weaknesses in the data available across countries and from the use of different measures of horizontal inequality and different proxies for economic (and social and political) inequality in the extant literature.

### Cross-Cuttingness

As discussed above, the degree to which various social cleavages overlap and intersect has long been highlighted in the literature as a key factor in understanding democratic stability and breakdown. Cross-cuttingness measures may be calculated to estimate the intersection between any two distinct dimensions of social divisions such as ethnicity, language, religion, and, as recently suggested, culture (Desmet et al. 2015). To the extent that cross-cuttingnessness measures may also capture the relationship between ethnic and non-ethnic cleavages, including socio-economic class, horizontal inequality can be seen as one type of cross-cuttingness measure.

Despite the importance of cross-cuttingness to the literature, quantitative analyses have rarely used cross-cuttingness measures. Although measures were developed early on (Rae and Taylor 1970), until Selway (2011) cross-national data on crosscuttingness were limited.^17^ Here we use Selway’s formulation, which is a measure of statistical independence. In the context of ethnicity and religion, it tells us how much an individual’s religion will also tell us about her ethnicity.

Compared to other countries, the data for Mindanao suggest a low degree of crosscuttingness overall. Selway (2011)’s data, for instance, indicate that the world region with the lowest crosscuttingness values, Eastern Europe and the former Soviet Bloc, has a country average of 0.61, which is higher than all of the values shown in Table 3. In Mindanao, the data suggest that ethnicity and religion are more cross-cutting the lower the administrative level, and slightly less cross-cutting in 2000 than 2010.^18^ In other words, cleavages appear to be more reinforcing at the Mindanao level than at the municipal or barangay level. If Lipset and Rokkan (1967) are correct, then, data for Mindanao on cross-cuttingness points to ethnic division being worse on average the larger the administrative unit.

## Comparative Findings and Guidance for Researchers

We highlight four conceptually major points that emerge from the preceding analysis to which we believe researchers should pay more attention and that deserve further investigation. These points will not be altogether new for specialists of ethnic politics (although frequently ignored in quantitative analysis), but the empirical precision with which they can be made here allows for deeper theoretical understanding and, importantly, comparisons between measures.

### Sensitivity to Chosen Measure

The eight measures highlighted here capture conceptually distinct aspects of societal divisions. Thus, while they point to similar trends and patterns, it should not be surprising that they do not necessarily correlate well empirically. Table 4 illustrates with the correlation matrix for ethnic measures in 2000 at the municipal level. As the data show, polarization, fractionalization, and cultural (distance) fractionalization are well-correlated. Yet, the weak correlations for most measures support the commonsensical (though not consistently-practiced) view that measures should be chosen with reference to theory and specifically their fit with posited causal mechanisms (Posner 2004). Understanding the conceptual logic underpinning a measure then is crucial as measures are not all interchangeable.

**Table 4:** Correlation among measures of ethnic divisions (municipalities, 2000 census, n = 375)

	Simple proportions (Moro)	Fractionalization	Cultural fractionalization	Polarization	Segregation (Moro–Lumad/Settler)	Intermarriage index (Moro–Settler)	Horizontal inequality GCOV	Cross-cuttingness
Simple proportions (Moro)	1.00							
Fractionalization	−0.23	1.00						
Cultural fractionalization	−0.24	1.00	1.00					
Polarization	−0.24	0.99	0.99	1.00				
Segregation (Moro–Lumad/Settler)	−0.23	0.24	0.24	0.24	1.00			
Intermarriage index (Moro–Settler)	−0.02	−0.39	−0.38	−0.37	−0.27	1.00		
Horizontal inequality GCOV	−0.19	0.76	0.76	0.75	0.20	−0.33	1.00	
Cross-cuttingness	0.14	−0.37	−0.36	−0.34	−0.25	0.57	−0.34	1.00

As we round to two decimal places, some measures appear perfectly correlated even though in actuality they are not

A secondary implication of measure distinctiveness is that more than one measure may be required to convey the multi-faceted character of divisions. If, for example, theory suggests both spatial and social distance between groups matter, then both segregation and cultural (distance) fractionalization should be considered. We should not assume any one measure will capture the full complexity of ethnic divisions.

In order to select an appropriate measure, researchers will need to specify the mechanism, that is the causal pathway through which ethnic divisions lead to particular outcomes. A key challenge often overlooked, however, relates to potential micro–macro disjunctions between measures and mechanisms. In the quantitative political economy literature, for instance, three prominent mechanisms rely on individual-level logics: (1) a preferences logic: coethnics share tastes, for instance, for particular policies or public goods or “favor” coethnics and “disfavor” non-coethnics due to prejudice or discrimination (Chandra 2001); or (2) a social connections logic: coethnics have stronger intragroup than intergroup ties and these facilitate ingroup reciprocity and accountability; or (3) a technology logic: coethnics share cultural characteristics such as language, traditions, and values and this promotes efficient cooperation (Habyarimana et al. 2007).

Yet, in marked contrast with the methodologically individualist logics behind these mechanisms, it is an easily-overlooked but important fact that all of these measures are constructed at the aggregate level. Thus, they do not directly capture individual-level preferences, connections, or characteristics, making it difficult to test these micro-logics with these measures. Further, we know that the link between micro-motives and macro-outcomes is not obvious (Schelling 1978) and that different aggregation techniques can produce radically different outcomes (Arrow 1950). Yet, the literature on these measures too rarely specifies the process linking the micro and macro levels or the theoretical justification behind the different aggregation techniques implicit in each measure. Researchers should consider then whether these individual-level logics and aggregation assumptions are appropriate for their theories. Indeed, there is no reason why measures of ethnic divisions need be based on an individual-level logic. One alternative approach, for instance, is proposed by Cederman et al. in theorizing ethnic civil war as the result of group-level logic: the larger an ethnic group is that is excluded from state power, the stronger its motivation to organize and rebel (Cederman and Girardin 2007). They construct a new measure, not reliant on aggregating individual characteristics, to match the group-level logic of their mechanism.

### Sensitivity to Categorization

The question of which ethnic categories should be used to construct measures of ethnic diversity has been the topic of considerable scholarly debate (Posner 2004; Fearon 2003; Alesina et al. 2003b; Chandra 2009). The case of Mindanao illustrates that the answer is often open to debate and may be multi-dimensional, even in severely divided societies. Choosing one set of categories over another in analysis thus requires both illumination and justification.

A common choice facing researchers is between using “census enumerated” and “politically salient” categories.^19^ The decision is consequential: in Mindanao, for instance, fractionalization values for 2010 are significantly higher using census-enumerated categories than politically salient ones: 0.89 (ethnic) and 0.58 (religious) compared with 0.53 and 0.38.

Here we have focused on politically salient ethnic and religious categories, i.e., “the groups that are actually doing the competing over policy, not the ones that an ethnographer [or census enumerator] happens to identify as representing distinct cultural units” (Posner 2004). Alternatively, one might argue that the ethno-religious identities and affiliations listed in the census better capture relevant categories, for instance, because they better reflect the categories locally-identified as relevant for official recording and thus salient to state-society relations. Or, that they are better because categories classified as “politically salient” might in fact be endogenous to the very outcomes one is trying to analyze.

But using census-enumerated categories is in fact less straightforward than it might seem as how precisely to interpret them is problematic. Take Mindanao. The 2000 and 2010 Philippine censuses include two relevant questions. One asks respondents to identify their religious affiliation and a second asks for “ethnicity by blood.” In 2000, 83 religious affiliations and 145 ethnic affiliations are classified, while 2010 enumerates 98 and 182 respectively.^20^ Variation in the categories listed from year to year then presents one challenge, particularly for “ethnicity by blood.” Only 106 ethnic categories are shared across the two censuses. Moreover, while the 2010 list is longer, it is not a more developed version of the 2000 list as 39 categories included in 2000 are not included in 2010. A second challenge is that the ethnic categories included are not all mutually exclusive. The two largest groups, Bisaya/Binisaya (22.93 %) and Cebuano (18.15 %), for instance, are sometimes treated as synonymous, while other census enumerated categories are considered by anthropologists to be subgroups of other enumerated groups (e.g., Tagabawa and Bagobo, respectively) (Masinaring 2011). Finally, not all enumerated ethnic categories are of the same type: Bisaya/Binisaya and Cebuano both generally refer to a Visayan language and a regional identity, while many other groups enumerated are indigenous peoples (e.g., Magbekin/Magbukon/Magbukun) and others are distinguished primarily by region (e.g., Capizeño).

In short, given the categories enumerated in the census, the same respondent could have “correctly” selected multiple categories; the choice of category from among “correct” alternatives then does not reveal an obvious and objectively verifiable ethnic group “by blood.” Rather, the sort of diversity shown in the Philippine census reinforces the ethnic politics constructivists’ point that ethnic identifications are “contingent, fuzzy, and situational” (Fearon 2003). The ethnic information reported in the census should not then be understood and used by researchers as objective evidence of the ethnic structure of society, but rather as one snapshot of an ever-evolving, complex, and socially-constructed system (see Nobles 2000).

Yet even if we accept that politically salient categories are superior to census-enumerated ones, one might still disagree with the politically salient categories as classified here. To heed our own recommendation to illuminate the categorization methodology, Table 5 lists those census-enumerated groups (in 2010) that we reclassified as either Moro or Lumad, i.e., the politically salient categories in Mindanao. All groups not classified as Moro or Lumad were deemed Settler groups. Notwithstanding this transparency, some of our choices are still debatable. For instance, with respect to the ethnic dimension, the Moro category could be understood as nested within the Lumad category as the latter refers to groups indigenous to Mindanao, while the former refers to groups indigenous to Mindanao that are Muslim. Here we treat the two as distinct given their distinct political salience. Indigeneity also is not an obvious or objectively clear status: recognition as an indigenous group is socially constructed and can be highly politicized, for instance providing access to land and political representation (Forte 2013; Flesken 2013; Gisselquist 2005). Here we aim to mirror as closely as possible the commonly understood indigenous status of each group—which is distinct from whether a group is “native” to Mindanao. For instance, Cotabateño Chavacano refers to a dialect of Chavacano, a Spanish-based creole language, with Cebuano, Hiligaynon, and Moro influences, originating in Cotabato City (Lewis et al. 2014). But while “native” to Cotabato City and with indigenous language influences, it is classified with the Settler category in our schema because it is not identified by our sources as “indigenous.”^21^

**Table 5:** Moro and Lumad categories in the 2010 census

Moro	Lumad^a^
Badjao, Bajao/Bajau, Iranon/Iranun/Iraynon, Jama Mapun, Kalagan, Kalibugan/Kolibugan, Maguindanao, Maranao, Molbog, Palawani, Sama Badajo, Sama Bangingi, Sama Laut, Sama/Samal, Sangil, Tausug, Yakan	Aromanen-Manobo, Ata, Ata/Negrito, Ata-Manobo, Bílaan/Blaan, Bagobo, Bagobo-Tagabawa, Banwaon, Bukidnon, Clata/Klata, Diangan, Dibabawon, Dibabeen Mulitaan, Dibaben, Direrayaan, Guiangan, Higaonon, Ilianen, Isoroken, Kailawan/Kaylawan, Kamiguin, Kirentenken, Lahitanen, Lambangian, Langilan, Livunganen, Mamanwa, Mandaya, Mangguangan, Manobo, Manobo-Blit, Manobo-Dulangan, Mansaka, Manubo-Ubo/Manobo-Ubo, Matigsalog/Matigsalug, Obu-Manuvu/Ubo-Manobo, Pulangien/Pulangiyen, Subanen/Subanon/Subanun, Tíboli/Tboli, Tagabawa, Tagakaulo, Talaandig, Talaingod, Teduray/Tiruray, Tigwahanon, Tinananen

^a^We also conducted analysis using a looser definition of this category as “Lumad and other non-Muslim locals,” i.e., to include also non-Moro groups native to the region but not classified as indigenous peoples (e.g., Cotabateño, Cotabateño-Chavacano, Davao-Chavacano, Davaweño, Surigaonon, and Zambageño-Chavacano)

In sum, as these choices with respect to categorization are complex, consequential, and far from self-evident, researchers should not bury them in their analyses, but should instead cast light on and provide rationales for them.

### Sensitivity to Time

As we show above, substantial changes can be seen in some of the standard measures of ethno-religious divisions even within a relatively brief period such as a decade. Moreover, the temporal sensitivity observed here reinforces our first point on how consequential the choice of measure is: three measures (intermarriage, segregation, and horizontal inequality) imply divisions are improving, whereas four suggest they are worsening (fractionalization, polarization, cultural distance, and cross-cuttingness). In order to explore the extent of such changes, we analyzed the overall percentage change between 2000 and 2010 for each of our measures (*x*) at each level of analysis. Given that some values are zeros, we use the following formula:

$$Temporal\,sensitivity = \frac{{\mathop \sum \nolimits_{i = 1}^{n} \left| {x_{{2000_{i} }} - x_{{2010_{i} }} } \right|}}{{\mathop \sum \nolimits_{i = 1}^{n} x_{{2000_{i} }} }}_{ }$$

where *n* represents the number of barangays, municipalities, provinces, or regions depending on the level of analysis. 

The data show clearly that all measures are sensitive to time, but some more than others. For purposes of illustration, Table 6 shows the values at the Mindanao level. Similarly large—and larger—changes can be seen at other levels of analysis. As Table 6 suggests, the intermarriage index is most sensitive to time: in just a decade, the Moro–Settler intermarriage rate increased by almost 70 %. At the other end of the spectrum, the change in horizontal inequality of religious groups (GCOV) was only about 5 %. At the Mindanao level, the average change across the measures shown here is just over 13 %.

**Table 6:** Percentage changes in diversity measures between 2000 and 2010 for Mindanao at the municipality level

Intermarriage index (Moro–Settler)	67.24
Intermarriage index (Muslim–Christian)	51.02
Fractionalization (ethnicity)	15.35
Cultural fractionalization (ethnicity)	13.12
Simple proportion (Moro)	9.97
Polarization (ethnicity)	8.16
Simple proportion (Muslim)	7.54
Horizontal inequality GCOV (ethnicity)	6.92
Polarization (religion)	4.52
Cultural fractionalization (religion)	4.46
Fractionalization (religion)	4.46
Segregation index of dissimilarity (Moro–Lumad/Settler)	2.55
Segregation index of dissimilarity (Muslim–Christian/Other)	1.47
Crosscuttingness (ethnicity and religion)	1.18
Horizontal inequality GCOV (religion)	0.05

The research literature highlights a number of factors that may drive changes in diversity over time. First, migration both across and within countries will alter the demographic balance at the national and subnational levels. We think migration was a large factor in Mindanao as the region was embroiled in war during the month that the 2000 census was conducted causing widespread internal displacement. Second, individuals, for a number of reasons, may shift their identification or affiliations over time. This may be due to slow-moving structural forces such as modernization and secularization or to more intentional actions such as religious conversion or assimilationist desires. Third, the reclassification and redefinition of groups, for instance through the census bureau, may also play a role (Nobles 2000). Lastly, differential birth or death rates across groups may further influence their relative sizes over time, although the effect in just a decade is probably small relative to other factors.

Regardless of its causes, the variation over time documented here underscores problems with the use of some of the most commonly-used data on ethno-religious diversity. The data used in *Atlas Narodov Mira* (1964), for instance, is over 50 years old, while the data used in Alesina et al. (2003b) is about 20 years old on average, and we detect significant changes in just a decade. The lack of up-to-date data on ethnicity and religion poses a major challenge for research. Given the extent to which ethno-religious landscapes change over time, we should be much more careful about drawing conclusions with out-of-date data. We should be especially concerned about temporal variation in conflict-affected societies given the likelihood of displacement, as well as in analysis of areas where economic migration is high, such as cities in developing countries.

### Sensitivity to Space

The data presented above unambiguously illustrate that divisions manifest differently across different administrative levels in Mindanao. We should not expect national-level cleavages to be reflected, *even on average*, at the local level. Moreover, there is a clear empirical pattern in Mindanao: as the level of aggregation declines, measures either rise or fall but generally do so monotonically. Simple proportions, the intermarriage index, and cross-cuttingness each tend to increase as the spatial unit gets smaller; whereas fractionalization, cultural (distance) fractionalization, polarization, segregation, and horizontal inequality each tend to decrease. Although measures move in opposing directions the story they collectively tell for Mindanao is generally consistent: divisions appear deeper when measured at higher levels of aggregation. Our impression of the extent of a society’s divisions then may be highly sensitive to the spatial level at which we measure them.

In order to compare the relative sensitivity of each measure to the selection of the spatial unit, we use the following simple formula:

$$Spatial\,sensitivity = \frac{{\left| {\bar{x}_{level\,A} - \bar{x}_{level\,B} } \right|}}{{\bar{x}_{level\,A} }}$$

where $\bar{x}$ is the average of the measure of interest and *level A* and *level B* refer to the two administrative levels being compared. Table 7 provides illustrative data for the Mindanao-to-municipality and region-to-province comparisons for 2000. As it suggests, there is spatial sensitivity in all of our measures, but comparatively less for segregation (index of dissimilarity), simple proportions, and horizontal inequality (GCOV). The intermarriage index is most sensitive to the selection of the level of analysis.

**Table 7:** Percentage changes in diversity measures between different areal units (2000)

	Between Mindanao and the average municipality	Between the average region and the average province
Intermarriage index (Moro–Settler)	1352.98	27.02
Intermarriage index (Muslim–Christian)	1350.90	30.31
Crosscuttingness (ethnicity and religion)	85.13	9.69
Fractionalization (religion)	63.59	14.69
Cultural fractionalization (religion)	63.59	14.69
Polarization (religion)	62.53	13.63
Fractionalization (ethnicity)	56.68	13.28
Cultural fractionalization (ethnicity)	55.79	12.39
Polarization (ethnicity)	50.42	11.55
Simple proportions (Moro)	43.53	0.32
Simple proportions (Muslim)	42.34	0.27
Horizontal inequality GCOV (ethnicity)	33.29	7.33
Horizontal inequality GCOV (religion)	31.10	4.24
Segregation index of dissimilarity (Moro–Lumad/Settler)	26.56	2.77
Segregation index of dissimilarity (Muslim–Christian/other)	25.56	2.15

The spatial variation observed here highlights several issues for researchers. First, researchers interested in the effects of ethnic divisions should take note of the modifiable areal unit problem (MAUP) (Openshaw 1983). The MAUP, a well-known issue among geographers, describes “the sensitivity of analytical results to the definition of [spatial] units for which data are collected” (Fotheringham and Wong 1991). It comprises two inter-related effects: a scaling effect, illustrated here, where the number (i.e., size) of units matters for inferences, and a zoning effect, where the boundaries (or shape) of the units matter. The MAUP then underlines the importance of selecting the appropriate unit of analysis using theory rather than the availability of data. We need to pay careful attention to the theoretical mechanism linking divisions to the outcome we are interested in explaining. The areal unit should be selected at the same level that theory posits the mechanism produces its effect. Generally, we would expect theory to suggest an areal unit that has political, social, or economic significance such as those defined by electoral or administrative boundaries.

Researchers then should rely on theory to describe the causal pathway and predict the level at which the outcome of interest will be observed. Take one well-known example. A commonly-posited theoretical mechanism for why ethnic diversity undermines public good provision is that non-coethnics have conflicting preferences that they express through voting (Alesina et al. 1999). Consequently, we would expect to see an effect at the level at which elected officials have discretion over the public good in question. For instance, if school budgets are decided at the state level, not the municipal level, we should then see a relationship between measures of ethnic division and school budgets at the state level, but not necessarily the municipal level. It is worth noting a secondary implication of this voting logic: electoral institutions will likely mediate the effects of conflicting ethnic preferences. It would be circumspect then for researchers to control for electoral institutional design as proportional representation systems may produce different effects to majoritarian systems.

Second, especially because so much research on the impact of ethnic divisions takes place at the cross-national level, analysts should beware the ecological fallacy. We should not assume that inferences drawn at the macro-level hold at the micro-level. For instance, countries that appear highly-conflicted by ethno-religious differences at the national level may either be harmonious or have different drivers of conflict at sub-national levels. In the context of our case, although ethno-religious divisions appear deeply-inscribed at the Mindanao-level, they may not explain conflict observed at the municipal-level, for instance. Indeed, finer-grained research suggests that other factors, notably clan rivalries, may be at work (Torres 2014). Similarly, research on the under-provision of public goods generally finds a negative correlation with ethnic diversity using national level data. Yet, recent work by Gerring et al. (2015) shows ethnic divisions may in fact be positively correlated with public goods provision at the *subnational* level.

Finally, the differences between subnational levels in measures of ethnic divisions observed here suggest researchers should also pay attention to the *spatial organization* of ethnic groups within society. Countries with the same ethnic divisions scores at the national level may nonetheless experience different political, social, and economic outcomes depending on the subnational configuration of ethnic groups in each. A small but growing number of researchers are finding settlement patterns within the unit of analysis matter for outcomes such as quality of government (Alesina and Zhuravskaya 2011), trust (Robinson 2015), and civil conflict (Matuszeski and Schneider 2006). In Mindanao, we find the island group does not appear so deeply-divided when we look at average scores of ethnic divisions at the barangay level. Yet if we considered the spatial organization of these barangays within Mindanao we would quickly realize the island is spatially segregated along ethnic and religious lines and may conversely conclude it is deeply-divided. Table 8 gives a simple example of two countries with three ethnic groups, each comprising equal shares of the national population. The two countries have the same national fractionalization scores. Yet in Country A, the same diversity shown at the national level is mirrored at the sub-national (state) level, whereas in Country B, each state is completely ethnically homogenous. Which country is more divided and prone to conflict?

**Table 8:** Example of spatial variation in fractionalization between two countries each with three groups of equal size and each comprising three subnational units (states)

	Country A	Country B
National-level fractionalization	0.67	0.67
Fractionalization in state a	0.67	0
Fractionalization in state b	0.67	0
Fractionalization in state c	0.67	0

The answer is not straightforward as the theoretical literature offers conflicting predictions. Some theorists argue territorial homogeneity is key to mediating ethno-national conflicts and advocate ethnic decentralization and federalism (Lijphart 1977). Conversely, others suggest such homogeneity (implying ethnic homelands) may heighten the risk of ethnic separatist conflicts (Toft 2001) or that heterogeneity is preferable as contact between ethnic groups will reduce prejudice and improve intergroup relations (Hewstone and Swart 2011). Notwithstanding the divergent predictions, these theories nonetheless all underscore the point that researchers should not ignore the potentially significant effect of ethnic settlement patterns in their analyses.

## Discussion and Conclusion

The hypothesis that ethno-religious divisions contribute to negative outcomes and poor quality of life in a variety of economic, political, and social domains has motivated an ever-expanding corpus of research. Yet this proposition is far from simple to test. We have sought to illustrate the complexity of conceptualizing and measuring divisions in ethnically and religiously diverse societies. Different bodies of research emphasize conceptually distinct aspects of divisions: from social structure to social distance to spatial distance between groups in society. Disaggregating the notion of division then is an important first step towards bringing conceptual coherence across disparate theoretical literatures. If ethnic divisions do indeed have negative impacts, disaggregation also has policy importance: if conflict results from cultural distance, for instance, review language policy; if it is spatial distance, consider housing policy. Yet the challenge is not only better conceptualization; it is also better theorization. Theories should clearly specify the causal pathway, that is the mechanism, through which ethnic divisions lead to particular outcomes. It is not theoretically sufficient, for instance, to state that conflicting preferences, weak cross-group ties, or cultural barriers cause undesirable outcomes without also specifying how precisely these factors are expected to do so.

Measurement should, logically, follow conceptualization and theorization. Data availability and calculation convenience should not mean either positing mechanisms to fit measures or disregarding the latter’s assumptions and limitations. We have highlighted the sensitivity of measures to the categorization methodology, the passage of time, and spatial variation. The latter two sensitivities suggest the dynamic character of divisions and we make two simple suggestions for better incorporating temporal and spatial dynamics in future research. For time, assuming longitudinal data exist, the change or rate of change in individual measures might be considered directly in analyses as either a causal factor or as an outcome. For space, researchers may wish to include one or more of the segregation measures in the analysis, in addition to their other measures of divisions, to capture or control for the variety of ethnic settlement patterns that may exist within society.

The stakes of getting concept and measure right are considerable. Policy-makers in diverse societies face a fundamental choice between preserving and eliminating ethnic differences, and the “diversity detriment” hypothesis risks misguiding a panoply of public policies if divisions are inappropriately conceptualized and measured (Gerring et al. 2015). At stake, for instance, are policies of institutional governance, nation-building and immigration, and electoral design: preservationists would advocate for federalism, multiculturalism, or proportional representation; eliminationists may alternatively campaign for partition, assimilation, or majoritarian voting (McGarry and O’Leary 1994). Finally, we also urge a rebalancing of the research agenda to consider the validity of a “diversity dividend” hypothesis (Gisselquist et al. 2015). “New” diversity, in the form of immigration, for instance, has been shown to be beneficial to the rejuvenation of the economies of advanced industrialized nations. Consistent and appropriate conceptualization and measurement of divisions across societal objectives then will be an imperative if policy-makers are to be able to fairly assess the merits and demerits of diversity.

## Footnotes

1 In particular, studies have raised questions about how measures can better capture hypothesized mechanisms (Montalvo and Reynal-Querol 2005), reflect multiple dimensions of division (Alesina et al. 2003b; Selway 2011), be based on politically relevant ethnic cleavages (Mozaffar et al. 2003; Posner 2004; Wucherpfennig et al. 2011), be sensitive to changes over time (Roeder 2001), and take seriously constructivist concerns about the endogeneity of ethnic identities (Chandra and Wilkinson 2008; Green 2013; Campos and Kuzeyev 2007).

2 Much of the literature refers interchangeably to ethnic divisions, diversity, and heterogeneity. In this article, we favor the term “divisions” as we see a subtle but important conceptual distinction between it and the latter two. Diversity and heterogeneity emphasize difference within society. Yet there are an infinite number of differences that might be enumerated and many of them have neither current nor historical social, political, or economic salience (Miguel 2004; Posner 2004). Divisions refer then only to “salient” differences, and it is for this reason we use data on politically-salient ethnic and religious categories to construct our measures.

3 Two measures, segregation (dissimilarity) and the intermarriage index, assume a two-group structure. Both, however, can be adapted for use with multiple groups although arguably sacrifice some interpretability in doing so.

4 Duclos et al. (2004) have also presented a multi-dimensional measure of polarization.

5 We note boundary change in itself may be indicative of societal divisions.

6 The discussion above also highlights the salience of clan divisions, which are not captured in the census.

7 It is worth noting that for four of the eight measures explored here—intermarriage, segregation, cross-cuttingness, and horizontal inequality—researchers face an important choice in how to report these values if their objective is to compare subnational rather than national units. In Mindanao, three religious groups (Muslim, Christian, Other) and three ethnic groups (Moro, Settler, Lumad) exist at the *national* level. However, not all three groups are present at all *subnational* levels. Some subnational units are perfectly homogeneous or else only two of the three religious or ethnic groups are present. Consequently, for subnational units where one or more of the national-level groups is wholly absent, researchers face a choice between calculating the measures using *all* the national-level groups (option A) or else using only the groups that exist in a particular subnational unit (option B). In perfectly homogeneous units, researchers strictly face no choice as the intermarriage, segregation, and cross-cuttingness scores cannot be calculated given the mathematical impossibility of dividing by zero. However, for horizontal inequality (which generates a value of zero for perfectly homogeneous units) and for the other three measures where at least two of the national-level groups exist, we believe option B can, in certain cases, lead to theoretically misleading outcomes when comparing subnational units. For instance, in calculating horizontal inequality in Mindanao, option B would imply that a community where only lowly-educated Muslims lived has the same value as a community where equal numbers of Muslims and Christians lived who had equal levels of high education. Consequently, we prefer to report option A, even though this has the undesirable practical effect of reducing the sample size.

8 Figures are calculated excluding observations for which religious and ethnic affiliation were not given by the respondent. Note the non-response rate was extremely small: only 2162 non-responses for religion and 5040 non-responses for ethnicity out of 21,903,385 individuals in the 2010 census data for instance i.e. less than 0.01 and 0.03 % non-response respectively.

9 The key differences are that the ELF is calculated as (1-HHI) and that the HHI uses the 50 largest firms while ELF generally is calculated using all ethnic groups identified.

10 In classifying language groups, Fearon (2003) and Desmet et al. (2009) rely on the *Ethnologue: Languages of the World* (see Lewis et al. 2014).

11 We report segregation scores assuming only two groups in society e.g. Muslims vs non-Muslims; Christians vs non-Christians. Although multi-group segregation measures exist (Reardon and Firebaugh 2002), their interpretation is not straightforward. We believe a better approach is to report a separate segregation score for each group of interest.

12 Evenness is only one of five conceptually distinct dimensions of segregation that sociologists originally distinguished to capture distinct patterns of settlement: the others are centralization, clustering, exposure, and concentration of groups. Segregation measures exist to capture each of these dimensions and scholars exploring theories that highlight the spatial organization of groups would do well to consider these as well.

13 Segregation measures are calculated using data from the next lowest level of government, thus they cannot be calculated below the municipality level here.

14 The most systematic analysis of horizontal inequality measurement has been conducted by Frances Stewart’s research program. In particular, Stewart et al. (2010) develops principles for evaluation of measures and recommends three best measures: GCOV, the group-weighted GINI (GGini), and the group-weighted Theil (GTheil). In our view, GCOV benefits from more straightforward interpretation (especially when compared to GTheil). For measures calculated at the group-level, see also Cederman et al. (2011).

15 Figures reported in Table 3 are based on the adult population only. As horizontal inequality is calculated here with respect to educational attainment including children may bias results if the age distribution of different ethnic groups differs.

16 See Footnote 7 for details.

17 Selway (2011) proposes three measures of cross-cuttingness: “subgroup fractionalization,” “crosscuttingness,” and “cross-fractionalization” (which is based on Rae and Taylor’s measure of cross-cuttingness). We use only the cross-cuttingness measure here, which in our view has the simplest interpretation.

18 As above, this is based on values for communities in which all three ethnic and all three religious groups salient at the national level are present. Hence, the sample size at the municipal and barangay levels is reduced.

19 Desmet et al. (2012) suggest another approach to categorization. They calculate linguistic diversity at different levels of aggregation and then test their predictive power against a range of political economy outcomes, arguing this method allows the data to identify the most salient cleavages.

20 We count “none” here as a religious affiliation.

21 More specifically, we adopted the following procedure: First, we recorded information available on all categories using eleven common sources. For categories with at least 0.009 % or more of the Mindanao population in either census, we then classified the category as Lumad, Moro, or Settler if two of these common sources provided consistent information. Additional sources were sought if information was inconsistent. For categories with less than 0.009 % of the Mindanao population, only one source was required. Second, targeted searches of online resources were then conducted for categories with insufficient information for classification until at least two sources were found. As possible, only information from official government sources, scholarly publications, established organizations, and newspaper articles was recorded; information from blogs or other websites was recorded only when no other information was found.
